# On-surface isostructural transformation from a hydrogen-bonded network to a coordination network for tuning the pore size and guest recognition†

**DOI:** 10.1039/d0sc05147k

**Published:** 2020-11-13

**Authors:** Dong-Dong Zhou, Jun Wang, Pin Chen, Yangyong He, Jun-Xi Wu, Sen Gao, Zhihao Zhong, Yunfei Du, Dingyong Zhong, Jie-Peng Zhang

**Affiliations:** MOE Key Laboratory of Bioinorganic and Synthetic Chemistry, School of Chemistry, Sun Yat-Sen University Guangzhou 510275 China zhangjp7@mail.sysu.edu.cn; School of Physics, State Key Laboratory of Optoelectronic Materials and Technologies, Sun Yat-Sen University Guangzhou 510275 China dyzhong@mail.sysu.edu.cn; National Supercomputer Center in Guangzhou, School of Data and Computer Science, Sun Yat-Sen University Guangzhou 510006 China

## Abstract

Rational manipulation of supramolecular structures on surfaces is of great importance and challenging. We show that imidazole-based hydrogen-bonded networks on a metal surface can transform into an isostructural coordination network for facile tuning of the pore size and guest recognition behaviours. Deposition of triangular-shaped benzotrisimidazole (H_3_btim) molecules on Au(111)/Ag(111) surfaces gives honeycomb networks linked by double N–H⋯N hydrogen bonds. While the H_3_btim hydrogen-bonded networks on Au(111) evaporate above 453 K, those on Ag(111) transform into isostructural [Ag_3_(btim)] coordination networks based on double N–Ag–N bonds at 423 K, by virtue of the unconventional metal–acid replacement reaction (Ag reduces H^+^). The transformation expands the pore diameter of the honeycomb networks from 3.8 Å to 6.9 Å, giving remarkably different host–guest recognition behaviours for fullerene and ferrocene molecules based on the size compatibility mechanism.

## Abstract 2

A hydrogen-bonded network on a Ag(111) surface can transform into an isostructural Ag(i) coordination network, giving drastically different host–guest recognition behaviours.

## Graphical abstract

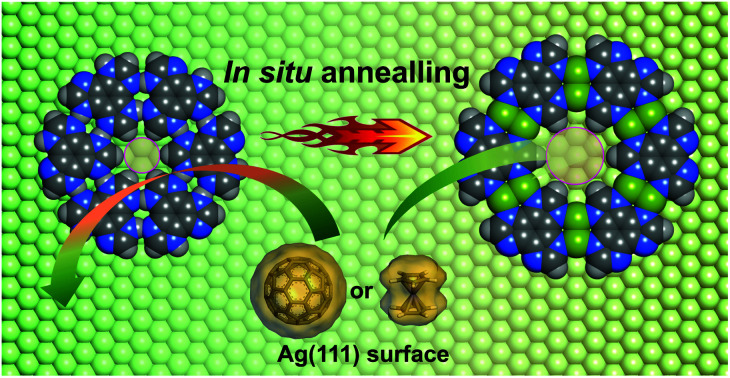

## Introduction

Surface-supported supramolecular structures, self-assembled from deposited molecules and/or metal atoms/ions *via* van der Waals interactions, hydrogen bonds, coordination bonds, *etc.*, have received continuous interest in recent years due to their straightforward and unique (supramolecular) chemistry, as well as great potential in functional molecular devices,^1–7^ although the synthesis difficulty, expense, scalability and applicability of these structures are still controversial. By applying heat, electric field, and/or light, or by further depositing other components, surface supramolecular structures may rearrange, and transform from hydrogen-bonded supramolecules into coordination ones, and even from supramolecules into macromolecules.^8–13^

Similar to three-dimensional (3D) solids, surface-supported 2D structures can have pores and serve as hosts for guest recognition.^14–21^ For example, deposition of linear dicarboxylic acid on a Cu surface could give hydrogen-bonded tetramers, and further deposition of Fe atoms could give ladder-type coordination networks with rectangular cavities, whose pore sizes can be tuned to accommodate one, two or three fullerene (C_60_) molecules, by using dicarboxylic acid with different lengths.^22^ Some 3D crystalline materials can be post-synthetically modified to achieve the fine-tuning of structures and properties, by reacting with external chemicals or under physical stimuli such as heat and light.^23–26^ Surface-supported 2D structures also have such reactivity. Note that the substrate can not only affect the surface structures but can also serve as the reactant.^27–31^ However, the initial and final 2D structures generally differ a lot so it is difficult to predict or tailor the structures and properties.

**Fig. 1:**
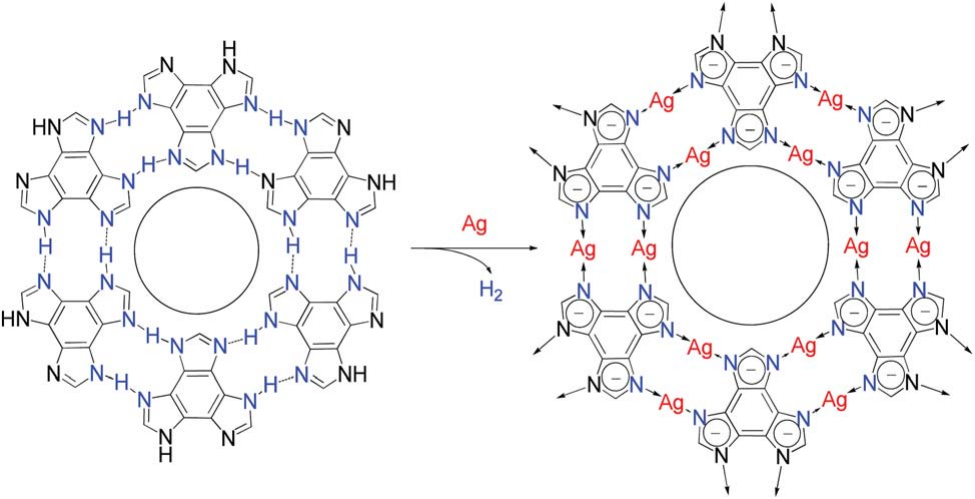
Topological transformation from a hydrogen-bonded network based on N–H⋯N bonds to an isostructural coordination network based on N–Ag–N bonds on Ag surfaces.

The protons on the ligands may be replaced by metal atoms (protons were reduced by metal atoms to hydrogen), but the connection modes between hydrogen-bonding and coordination-bonding are generally different, making it difficult to predict 2D structures before and after transformation.^28,32–34^ The linear coordination mode of monovalent coinage metal ions resembles those of strong hydrogen bonds, which has been well demonstrated in metal imidazolate frameworks.^35–37^ However, surface-supported coordination networks based on imidazole derivatives have not been reported, probably because imidazole derivatives and their metal complexes can seldom form planar supramolecular structures. We designed benzo[1,2-*d*:3,4-*d*′:5,6-*d*′′]trisimidazole (H_3_btim) as a rigid planar imidazole derivative suitable for forming 2D planar honeycomb-like networks connected by doubled N–H⋯N hydrogen bonds. Although in the bulk crystalline form the extremely small intralayer cavities of the staggered-stacking 2D honeycomb networks are inaccessible to any guest,^38^ and H_3_btim in the solution/solid state is difficult to react with metals or metal ions to form coordination compounds, we show here that hydrogen-bonded H_3_btim honeycomb networks on Ag(111) can react with metal surfaces and transform into isostructural coordination networks (Fig. 1), which facilely expands the pore size and drastically changes the guest recognition behaviors toward C_60_ and ferrocene (Fe(Cp)_2_) molecules.^39,40^

## Results and discussion

### Honeycomb-like hydrogen-bonded networks

**Fig. 2:**
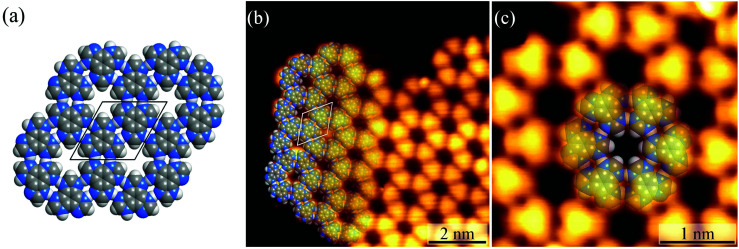
(a) The 2D hydrogen-bonded network in H_3_btim bulk crystals. (b) The large-scale (*U* = −0.2 V, *I* = 1 nA) and (c) high-resolution (*U* = −0.6 V, *I* = 100 pA) STM image of H_3_btim molecules on Au(111) surfaces. The unit cell is indicated in black/white in (a)/(b). Molecular models are superimposed on the respective image.

Under ultrahigh vacuum in scanning tunneling microscope (STM) equipment (*ca.* 10^−10^ mbar), H_3_btim can evaporate at 503 K. For comparison, under common vacuum (*ca.* 10^−4^ mbar) and ambient pressure, the evaporation temperature needs to be 623 K and 673 K, respectively.^38^ STM showed that the H_3_btim molecules deposited on Au(111) and Ag(111) surfaces form large-area, regular and ordered honeycomb-like patterns resembling that of hydrogen-bonded layer fragments of its crystal structure (Fig. 2 and S1†).^38^ The lattice period (*T*) and the diameter of the hole (*d*) of the honeycomb-like patterns were measured to be ∼12.0 Å and ∼3.8 Å, respectively, consistent with the crystallographic results (12.7 Å and 3.8 Å, Fig. 2a and b). In the high-resolution STM image (Fig. 2c), H_3_btim molecules were present as rounded triangular protrusions individually, and there is a hollow inter-space between two adjacent molecules, corresponding to N–H⋯N hydrogen bonds (Fig. 1). These results demonstrated that the honeycomb-like structures indeed interconnect through double N–H⋯N hydrogen bonds. Although similar surface-supported hydrogen-bonded honeycomb-like 2D structures have been reported,^41,42^ H_3_btim is the first example purely linked by N–H⋯N hydrogen bonds.

While H_3_btim molecules show the same self-assembled structure on Au and Ag surfaces, they possess distinctly different stabilities and reactiveness. By gradually increasing the substrate temperature, the honeycomb-like structures on Au(111) disappeared at 453 K (Fig. S2 and S3†), which is lower than that of bulk H_3_btim, indicating that the interaction between H_3_btim molecules and the Au(111) surface is weaker than the π–π interaction in the bulk H_3_btim crystal. The physisorption feature is also implied by the unchanged herring-bone reconstruction of the Au(111) surface underneath the self-assembled H_3_btim networks (Fig. S2 and S3†).

### Honeycomb-like coordination networks

**Fig. 3:**
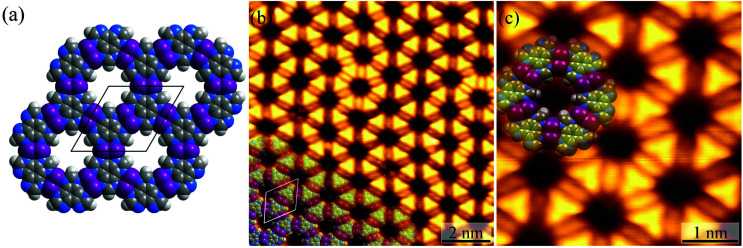
(a) MM simulated structure of [Ag_3_btim]. (b) The large-scale (*U* = 1.6 V, *I* = 100 pA) and (c) high-resolution (*U* = 0.08 V, *I* = 1.54 nA) STM image of [Ag_3_(btim)] on Ag(111) surfaces. The unit cell is indicated in black/white in (a)/(b). Molecular models are superimposed on the respective image.

For the Ag(111) surface, additional bright spots/rods appeared between the H_3_btim molecules, when the temperature was increased to 373 K, resulting in disordering of the honeycomb networks. The transformation completed at 423 K with the recurrence of ordered honeycomb networks (Fig. 3 and S4†). However, the measured *T* and *d* increased to ∼14.0 Å and ∼6.9 Å, due to the enlarged intermolecular spacing induced by intercalation of Ag atoms. To our knowledge, so far there has been no metal complex of H_3_btim or its deprotonated forms reported. We simulated the 2D honeycomb-like [Ag_3_(btim)] structure by the Molecular Mechanics (MM) method, giving a *T* of 14.4 Å and *d* of 6.8 Å, consistent with the measured values (Fig. 3). In the high-resolution STM image, the morphology of H_3_btim molecules transformed from a rounded triangle to a sharp one, corresponding to the redistribution of molecular orbitals by deprotonation. Between each pair of adjacent triangles, a fusiform spot appeared and can be assigned to the two Ag(i) ions connecting the btim^3−^ ligands (Fig. 3).^43–45^

It should be noted that the H_3_btim hydrogen-bonded network can fully cover the Ag(111) surface, but partial desorption of H_3_btim molecules from the surface occurred during the annealing process (Fig. S5†), and samples with an initial coverage varying from a submonolayer to monolayer could result in identical [Ag_3_(btim)] structures (Fig. S6†). The size of the [Ag_3_btim] networks on Ag(111) surfaces can be up to 200 nm (Fig. S5†), and an external metal source and continuous H_3_btim molecule deposition would benefit the growth of larger [Ag_3_(btim)] networks.^46^ Moreover, the reverse transformation from the coordination to hydrogen-bonded networks was not observed, being consistent with the irreversible hydrogen evolution process. Nevertheless, samples with both structures coexisting on the surface can be prepared by secondary deposition of H_3_btim molecules on the surface with [Ag_3_(btim)] (Fig. S7†).

### Host–guest recognition

**Fig. 4:**
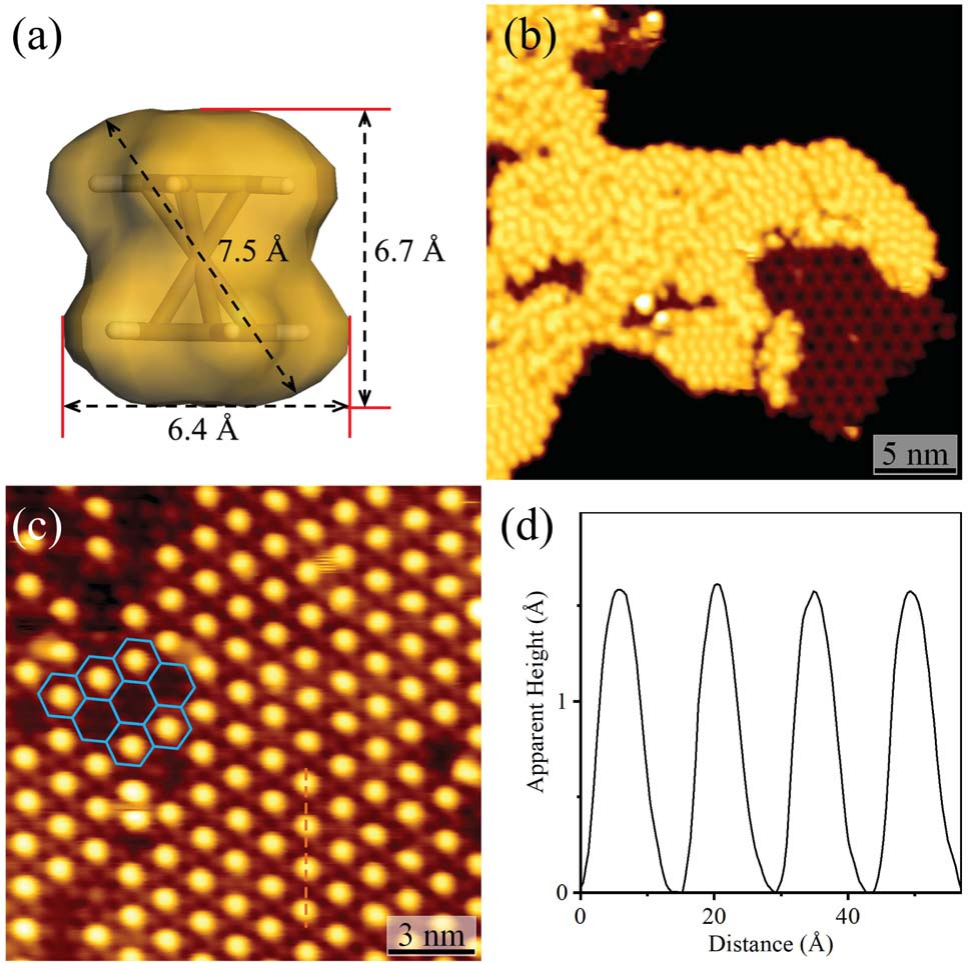
Adsorption of Fe(Cp)_2_ on H_3_btim networks or [Ag_3_(btim)] networks. (a) The molecular size and shape of Fe(Cp)_2_. (b) STM image of Fe(Cp)_2_ molecules on H_3_btim networks (*U* = −3.0 V, *I* = 10 pA). (c) STM image of Fe(Cp)_2_ molecules on [Ag_3_(btim)] networks (*U* = −2.0 V, *I* = 50 pA) with (d) the height profile along the orange line in Fig. 4c.

By using C_60_ or Fe(Cp)_2_ with different sizes as the guest (Fig. 4a and S8†), the host–guest recognition behaviours of the isostructural hydrogen-bonded and coordination networks were investigated. STM showed that the large C_60_ molecules aggregated periodically with a hexagonal pattern on bare Ag(111) and on the hydrogen-bonded H_3_btim network assembled on Ag(111) (Fig. S9†). The lattice period of C_60_ molecules was measured to be ∼9.5 Å, being similar to the values (10.2 Å) on other metal substrates,^47^ and close to the molecular size (9.8 Å), but obviously smaller than that of the H_3_btim network on Ag(111) (12.7 Å). On [Ag_3_(btim)], low-concentration C_60_ molecules located randomly, but they showed very similar heights with a difference of less than 0.2 Å (Fig. S10†), and the shortest intermolecular distances are ∼14.0 Å (Fig. S10†), being consistent with the *T* value of [Ag_3_(btim)], which indicates that they are located at the holes. For comparison, C_60_ molecules on H_3_btim showed a large height difference of *ca.* 0.5 Å (Fig. S9†), indicating that they locate at different environments. Nevertheless, high-concentration C_60_ molecules tend to aggregate on the Ag(111) surface (Fig. S11†).

On the H_3_btim hydrogen-bonded networks, Fe(Cp)_2_ molecules distributed randomly with adjacent distances of 6.5–10.5 Å (Fig. 4b and S12†), far shorter than the *T* value of H_3_btim. By contrast, on [Ag_3_(btim)], Fe(Cp)_2_ molecules showed large-area regular patterns (*T* ∼ 14.0 Å) matching well with those of the holes of the host (Fig. 4c and d). These indicate that the larger holes of [Ag_3_(btim)], compared with H_3_btim, are suitable for accommodating Fe(Cp)_2_.

### Computational calculations

**Fig. 5:**
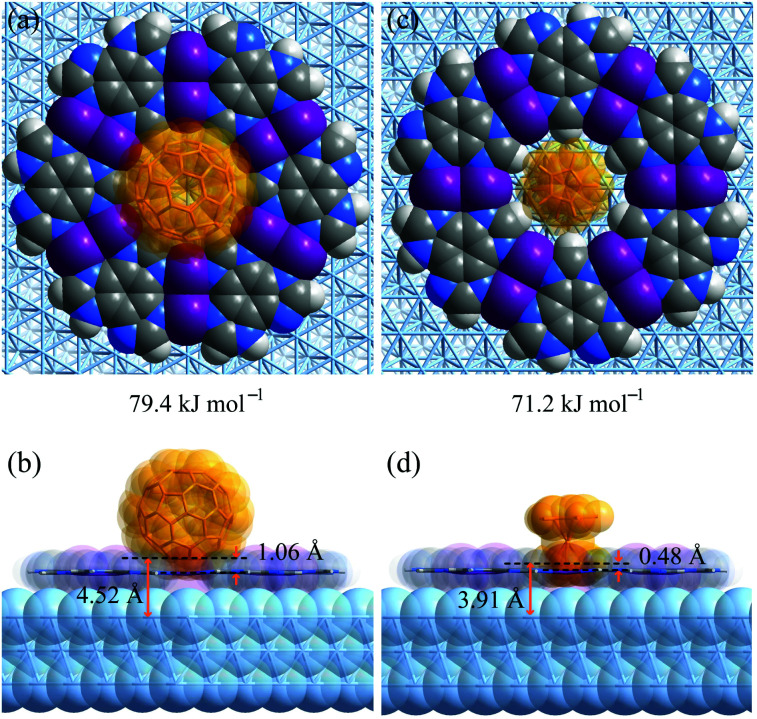
DFT simulated primary host–guest structures on Ag(111) supported [Ag_3_(btim)] networks. (a and b) C_60_. (c and d) Fe(Cp)_2_. (a and c) Top views. (b and d) Side views.

To explain the different host–guest recognition behaviours, the adsorption enthalpies (Δ*H*) of C_60_ and Fe(Cp)_2_ at typical environments were calculated by density functional theory (DFT) simulations (Fig. 5 and S13–S17†). For C_60_ on H_3_btim, Δ*H* follows imidazole ring (−52.1 kJ mol^−1^) < phenyl ring (−43.4 kJ mol^−1^) < hole (−40.1 kJ mol^−1^) < hydrogen bonds (−31.5 kJ mol^−1^). For Fe(Cp)_2_ on H_3_btim, Δ*H* follows hydrogen bonds (−27.7 kJ mol^−1^) < hole (−25.2 kJ mol^−1^) < phenyl ring (−11.9 kJ mol^−1^) < imidazole ring (−7.9 kJ mol^−1^). Because C_60_ will be too crowded to fully occupy the primary adsorption site (imidazole ring), and Fe(Cp)_2_ exhibits little difference between the primary and secondary adsorption sites, these guests cannot form an ordered host–guest arrangement on H_3_btim. In contrast, the primary adsorption sites for C_60_ and Fe(Cp)_2_ on [Ag_3_(btim)] networks are both on the holes, and their Δ*H* values (−79.4 kJ mol^−1^ and −71.2 kJ mol^−1^) are much lower than those of the secondary adsorption sites (−56.9 kJ mol^−1^ for C_60_ on the phenyl ring and −42.8 kJ mol^−1^ for Fe(Cp)_2_ on the double N–Ag–N bonds, respectively). Therefore, C_60_ and Fe(Cp)_2_ tend to locate on the holes of [Ag_3_(btim)] networks.

Comparison of the host–guest structures on the holes clearly explained the differences of recognition behaviors based on the size mechanism. On [Ag_3_(btim)], the distances from the deepest inserting atom of C_60_ to the coordination network plane and the Ag(111) surface are 1.06 Å and 4.52 Å (Fig. 5a and b), which are significantly shorter than and slightly longer than the sum of the van der Waals radii of the corresponding atoms (C 1.7 Å, Ag 2.2 Å), *i.e.,* 3.4 Å and 3.9 Å, respectively. This means C_60_ can partially insert into the hole and cannot touch the Ag(111) surface. By contrast, these distances are 0.48 Å and 3.91 Å for Fe(Cp)_2_ on [Ag_3_(btim)], indicating that the smaller Fe(Cp)_2_ molecule can fully insert to touch the Ag(111) surface (Fig. 5c and d). Therefore, the Δ*H* difference of Fe(Cp)_2_ and C_60_ on [Ag_3_(btim)]@Ag(111) is far less than those of their molecular sizes and boiling points. For comparison, these distances for C_60_/Fe(Cp)_2_ on H_3_btim@Ag(111) are 2.21/2.14 Å and 5.66/5.61 Å, respectively (Fig. S13†), consistent with relatively high Δ*H* values.

The adsorption of C_60_ (−75.2 kJ mol^−1^) on Ag(111) is slightly weaker than on the hole of [Ag_3_(btim)] (Fig. S18†). On Ag(111), C_60_ molecules can approach each other to furnish close and strong intermolecular interactions (−13.4 kJ mol^−1^ with a center–center distance of 9.9 Å, Fig. S19†). On the holes of [Ag_3_(btim)], intermolecular interactions between adjacent C_60_ molecules are much weaker (−3.9 kJ mol^−1^ with a center–center distance of 14.4 Å, Fig. S19†). Therefore, two or more C_60_ molecules tend to aggregate on Ag(111) rather than periodically locate on the holes of [Ag_3_(btim)]. For comparison, the adsorption of Fe(Cp)_2_ on Ag(111) (−21.5 kJ mol^−1^, Fig. S18†) is much weaker than on the holes of [Ag_3_(btim)] (−71.2 kJ mol^−1^).

## Conclusions

In summary, we investigated the self-assembly and reactivity of a high-symmetry imidazole derivative on Au(111)/Ag(111) surfaces, and achieved *in situ* transformation from a hydrogen-bonded network to an isostructural coordination network and fine tuning of pore sizes. Moreover, we directly observed the vastly different adsorption and recognition behaviors of guest molecules on these 2D porous networks, demonstrating the important role of pore size tailoring. These results can provide a new clue for the preparation and property modulation of new 2D materials, which have potential for energy conversion and storage.

## Experimental

Fullerene (C_60_) and ferrocene (Fe(Cp)_2_) were brought from Alfa Aesar (purity: 99.5%), and benzo[1,2-*d*:3,4-*d*′:5,6-*d*′′]trisimidazole (H_3_btim) was synthesized according to the literature,^38^ and they were used without further purification.

STM measurements were carried out on an Omicron low-temperature STM system with a base pressure below 1 × 10^−10^ mbar. Clean metal substrates were obtained by repeated Ar^+^ sputtering (at 298 K for 12 min) and annealing (at 750 K for 30 min). All the STM images were taken in the constant-current mode by using electrochemically etched tungsten tips with the samples cooled down with liquid nitrogen to 78 K. All given voltages refer to the bias on samples with respect to the STM tip.

A quartz crucible containing H_3_btim microcrystalline powders was put in the sample injection chamber. The chamber was degassed to *ca.* 10^−8^ mbar and the crucible was heated to 473 K for several hours to remove impurities in H_3_btim. Subsequently, the crucible was heated at 503 K, and the sublimated H_3_btim molecules travelled through the molecular beam epitaxy (MBE) system to deposit onto the substrate surface (at 298 K). The obtained sample was then transferred from the preparation chamber to the STM chamber without exposure to air. The method for deposition of C_60_ molecules on the H_3_btim honeycomb network was the same as for deposition of H_3_btim, except that the sublimation/crucible temperature was 623 K and the deposition/substrate temperature was 273 K. Due to the easy evaporation/sublimation, Fe(Cp)_2_ powders were stored in a tin-foil crucible (diameter: 1.5 mm) rather than the quartz crucible. To start depositing Fe(Cp)_2_ molecules, a home-made tin-foil crucible was transferred to the preparation chamber, and then put at a place near the target substrate (<200 K).

Molecular mechanics (MM) simulations were performed to obtain simulated a honeycomb-like [Ag_3_(btim)] structure by using the Forcite module in the Materials Studio 5.5 package. The initial configurations were produced by replacing H atoms of monolayer H_3_tim with Ag atoms. Structural optimization was based on the universal forcefield (UFF); all atoms and cell parameters were regarded as variable and *Q*_eq_ partial charges were employed. The cutoff radius was chosen as 18.5 Å for the LJ potential. Density functional theory (DFT) calculations were performed to obtain the adsorption enthalpies between hosts and guests by using the Vienna Ab-initio Simulation Package (VASP) package.^48,49^ The Perdew–Burke–Ernzerhof (PBE) form of the generalized-gradient approximation (GGA) was used to treat the electronic exchange correlation. The energy cutoff was chosen to be 520 eV and the system was relaxed in the self-consistency accuracy of 10^−4^ eV. All atoms in the unit cell were minimized by the conjugate gradient method until the force on each atom was less than 0.01 eV Å^−1^. The *Γ* point was used to integrate the Brillouin zone by the gamma-centered sampling method.

Since the H_3_btim structure is incommensurate with the Ag(111) surface, an extremely large supercell is required in order to match both of them, making it hard to handle during DFT calculations. For better comparison of the binding energy differences between the H_3_btim and [Ag_3_(btim)] host–guest systems, non-periodic networks on the Ag(111) surface were used. When considering the effect of the Ag(111) surface below the holes, a 7 × 7 × 7 Ag(111) supercell was used to construct the surface slab and a hexamer of H_3_btim or the corresponding [Ag_3_(btim)] fragment was built above the Ag(111) surface. Moreover, the Ag(111) surface slab built with 3 layers of Ag atoms and a vacuum space of 25 Å was employed to avoid interactions between the top and bottom surfaces. The topmost 2 layers were relaxed during optimization, while the remaining 1 layer was kept fixed to mimic the bulk.^50–52^ The vdW-DF2 method^53^ was employed to evaluate the van der Waals (vdW) effect in all calculations. Three potential binding sites (hydrogen bonds, the phenyl group, and the imidazole group of H_3_btim, while double N–Ag–N bonds, the phenyl group, and the imidazolate group of [Ag_3_(btim)]) were selected to calculate the adsorption enthalpies. C_60_ and Fe(Cp)_2_ were initially placed 2.5–3.0 Å above the hosts, and then the whole host–guest systems were optimized to the lowest energy position. The adsorption enthalpy (Δ*H*) is defined as follows:Δ*H* = *E*_host+guest_ − *E*_host_ − *E*_guest_where *E*_host+guest_, *E*_host_, and *E*_guest_ are the energy of the final host–guest system, the pristine host, and the guest before adsorption in a vacuum environment, respectively.

## Conflicts of interest

There are no conflicts to declare.

## Supplementary Material

SC-012-D0SC05147K-s001
