# Supplementary material for: On-surface isostructural transformation from a hydrogen-bonded network to a coordination network for tuning the pore size and guest recognition
Source: Chem Sci. 2020 Nov 13;12(4):1272–7. doi: 10.1039/d0sc05147k (PMC8179111; doi:10.1039/d0sc05147k)
Supplement: SC-012-D0SC05147K-s001 [file SC-012-D0SC05147K-s001.pdf]

Electronic Supplementary Material (ESI) for Chemical Science.
This journal is © The Royal Society of Chemistry 2020

Electronic Supplementary Information (ESI) for

On-surface isostructural transformation from hydrogen-bonded to coordination network for tuning pore size and guest recognition

Dong-Dong Zhou,[‡a] Jun Wang,[‡b] Pin Chen,[‡c] Yangyong He,[b] Jun-Xi Wu,[a] Sen Gao,[c] Zhihao Zhong,[b] Yunfei Du,[c] Dingyong Zhong,[*b] and Jie-Peng Zhang[*a]

[a] MOE Key Laboratory of Bioinorganic and Synthetic Chemistry, School of Chemistry, Sun Yat-Sen University, Guangzhou 510275, China

[b] State Key Laboratory of Optoelectronic Materials and Technologies, School of Physics, Sun Yat-Sen University, Guangzhou 510275, China

[c] National Supercomputer Center in Guangzhou, School of Data and Computer Science, Sun Yat-Sen University, Guangzhou 510006, China

[‡] These authors contributed equally: Dong-Dong Zhou, Jun Wang, Pin Chen

*E-mail: dyzhong@mail.sysu.edu.cn; zhangjp7@mail.sysu.edu.cn

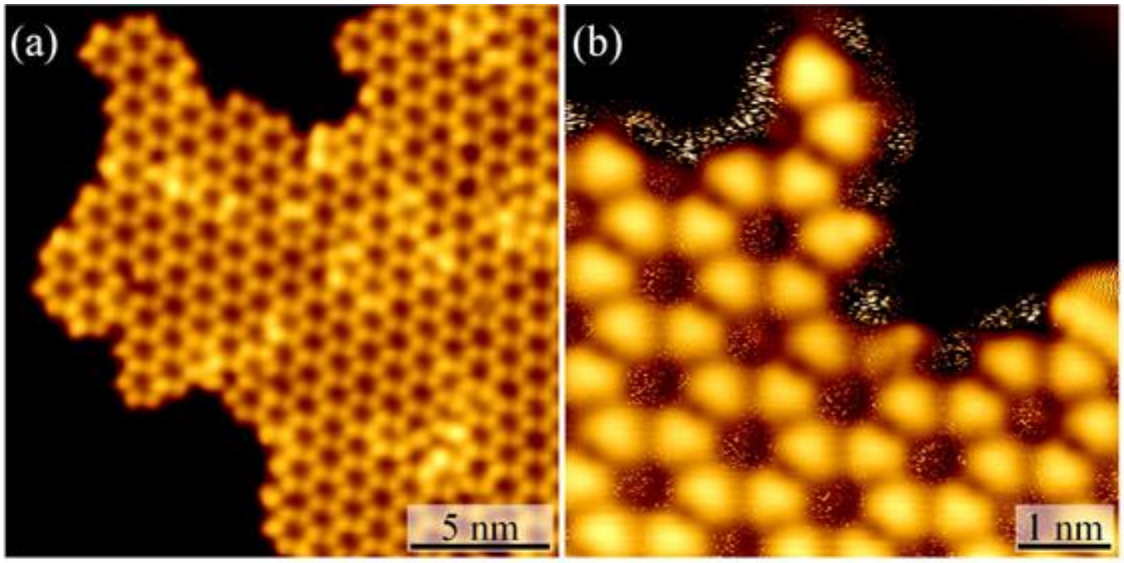

Fig. S1 The STM image of H_3btim molecules on Ag(111) surfaces in (a) large area (U = 2.2 V, I = 50 pA) and (b) small area (U = −0.1 V, I = 200 pA).

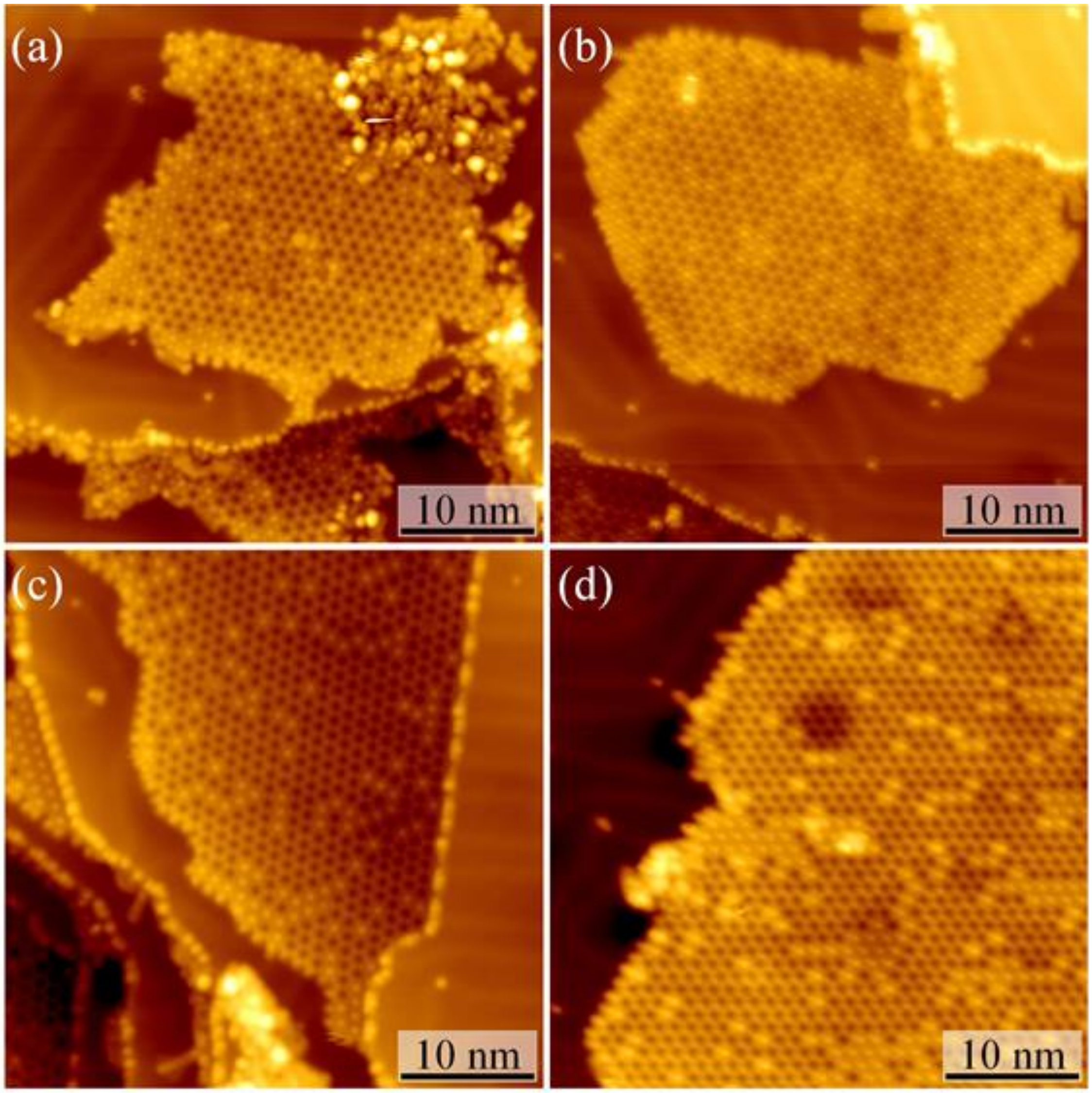

Fig. S2 The STM images of H_3btim molecules on Au(111) surfaces after annealing at (a) 373 K (U = 1.5 V, I = 30 pA), (b) 393 K (U = 2.0 V, I = 80 pA), (c) 413 K (U = 2.0 V, I = 20 pA) and (d) 433 K (U = 1.8 V, I = 30 pA).

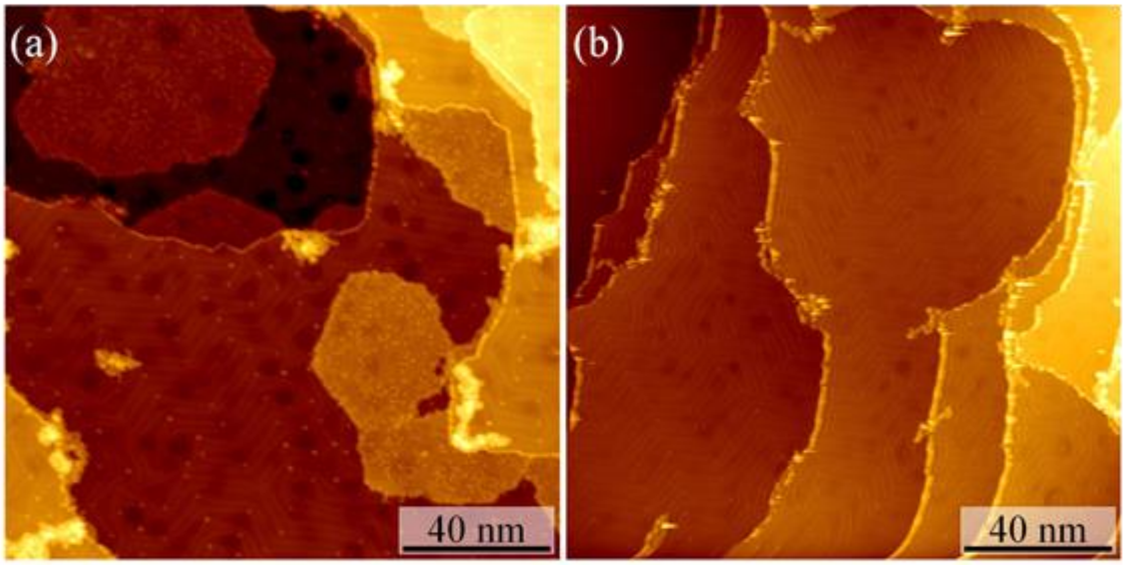

Fig. S3 The STM images of H_3btim molecules on Au(111) surfaces (near fault) after annealing at (a) 433 K (U = 1.8 V, I = 30 pA) and (b) 453 K (U = −2.0 V, I = 20 pA).

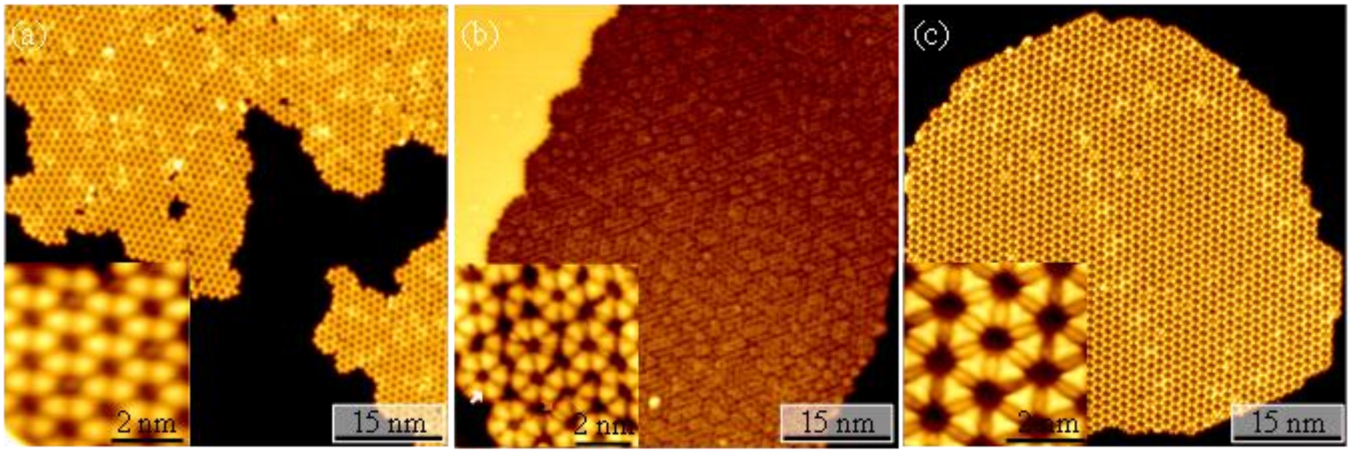

Fig. S4 The large-scale and the high-resolution (inset) STM images of H_3btim molecules on Ag(111) surfaces (a) before annealing (U = 2.2 V, I = 50 pA; inset: U = –0.4 V, I = 100 pA), and after annealing at (b) 373 K (U = 1.8 V, I = 50 pA); inset: U = –1 V, I = 800 pA) and (c) 423 K (U = 1.6 V, I = 100 pA; inset: U = 0.05 V, I = 1.54 nA). The hydrogen bonding and N–Ag–N bonding are marked by white and yellow arrow in Fig. S4b, respectively.

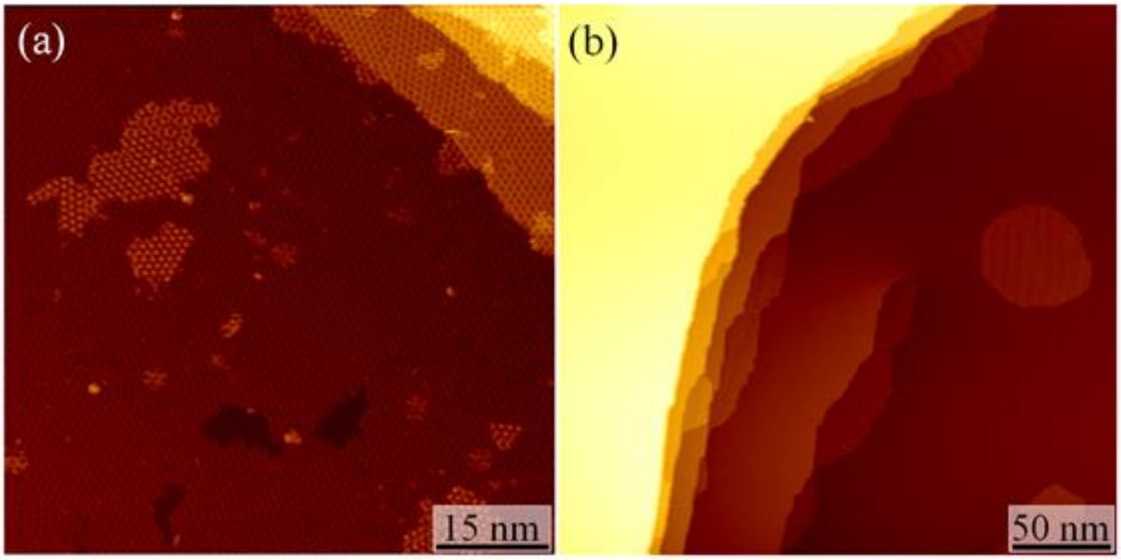

Fig. S5 The large-scale STM images of H_3btim molecules on Ag(111) surfaces (a) before annealing (U = −2V, I = 20 pA) and (b) after annealing (U = 2V, I = 20 pA).

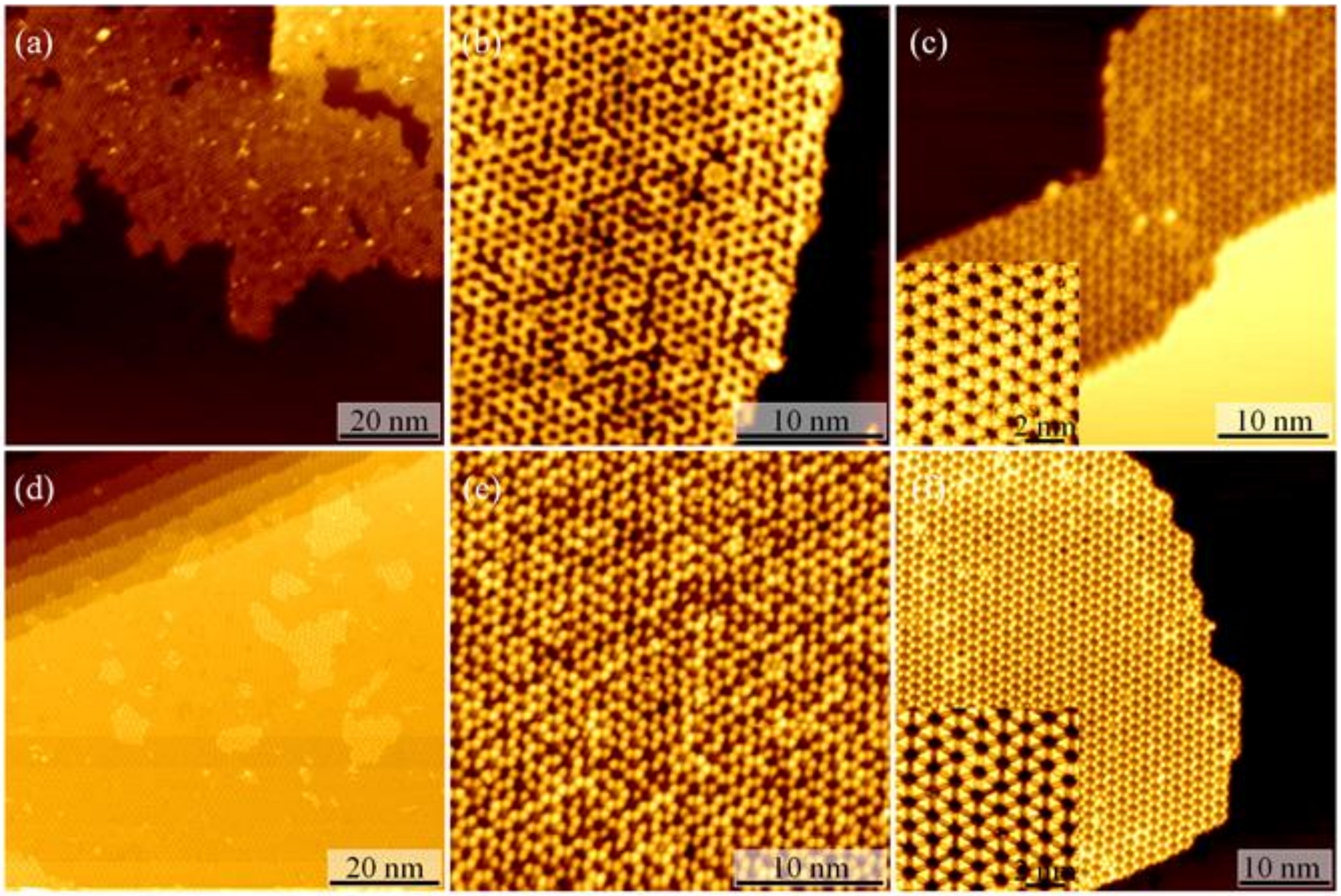

Fig. S6 The STM images of H_3btim molecules with different coverages on Ag(111) surfaces. (a, b, c) The low coverage case, (a) before annealing (U = –2.0 V, I = 50 pA), and after annealing at (b) 373 K (U = 1.5 V, I = 80 pA) and (c) 393 K (U = 1.7 V, I = 30 pA; inset: U = –0.3 V, I = 10 pA). (d, e, f) The high coverage case, (d) before annealing (U = –2.0 V, I = 20 pA), and after annealing at (e) 413 K (U = –2.0 V, I = 100 pA) and (f) 423 K (U = 1.5 V, I = 300 pA; inset: U = 1.6 V, I = 100 pA).

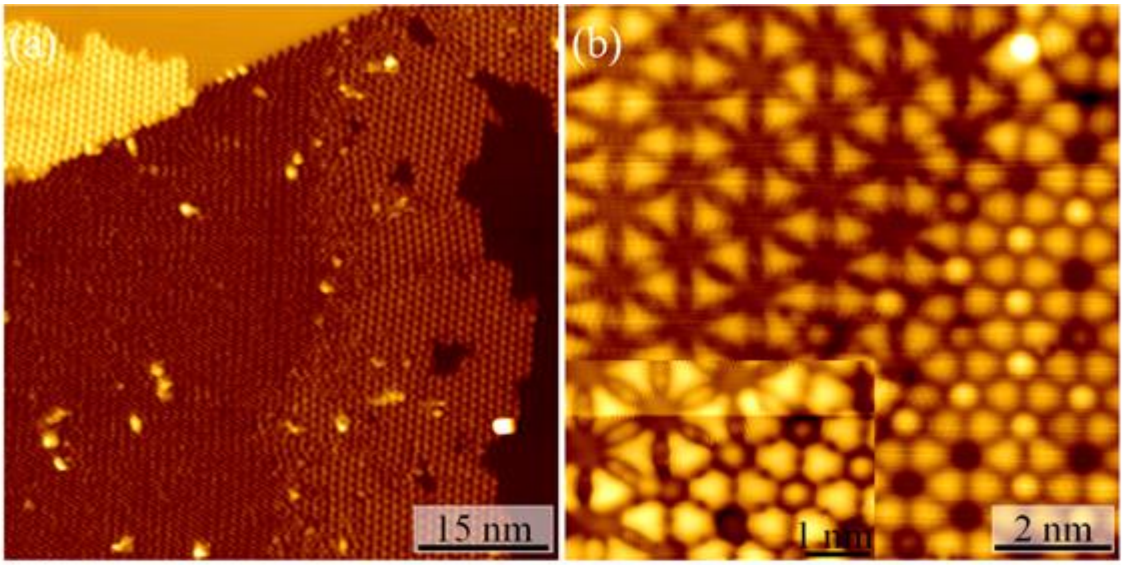

Fig. S7 The STM images of the redeposited H_3btim molecules on Ag(111) surfaces with [Ag_3(btim)]. (a) The large-scale (U = –2.0 V, I = 20 pA) and (b) the medium-scale (U = –0.2 V, I = 1.1 nA), inset: the high-resolution (U = 0.01 V, I = 1.6 nA).

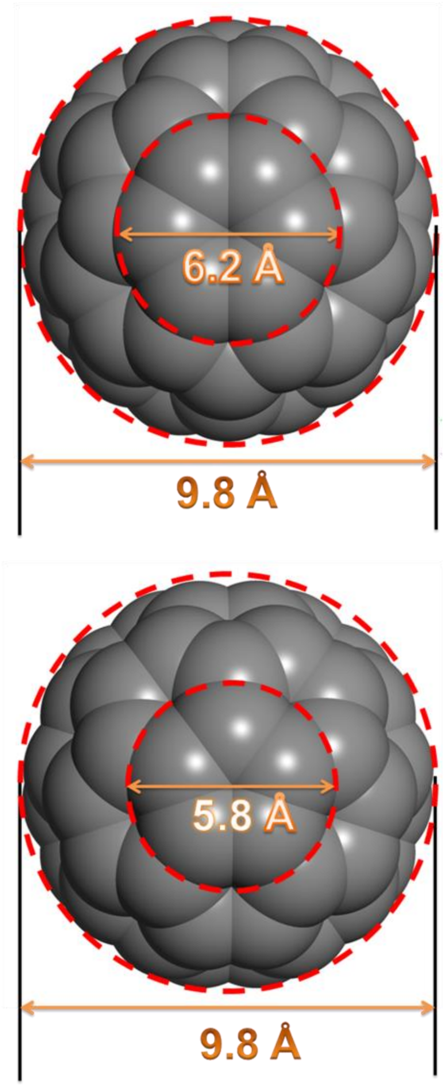

Fig. S8 The key size parameters for C_{60} molecules.

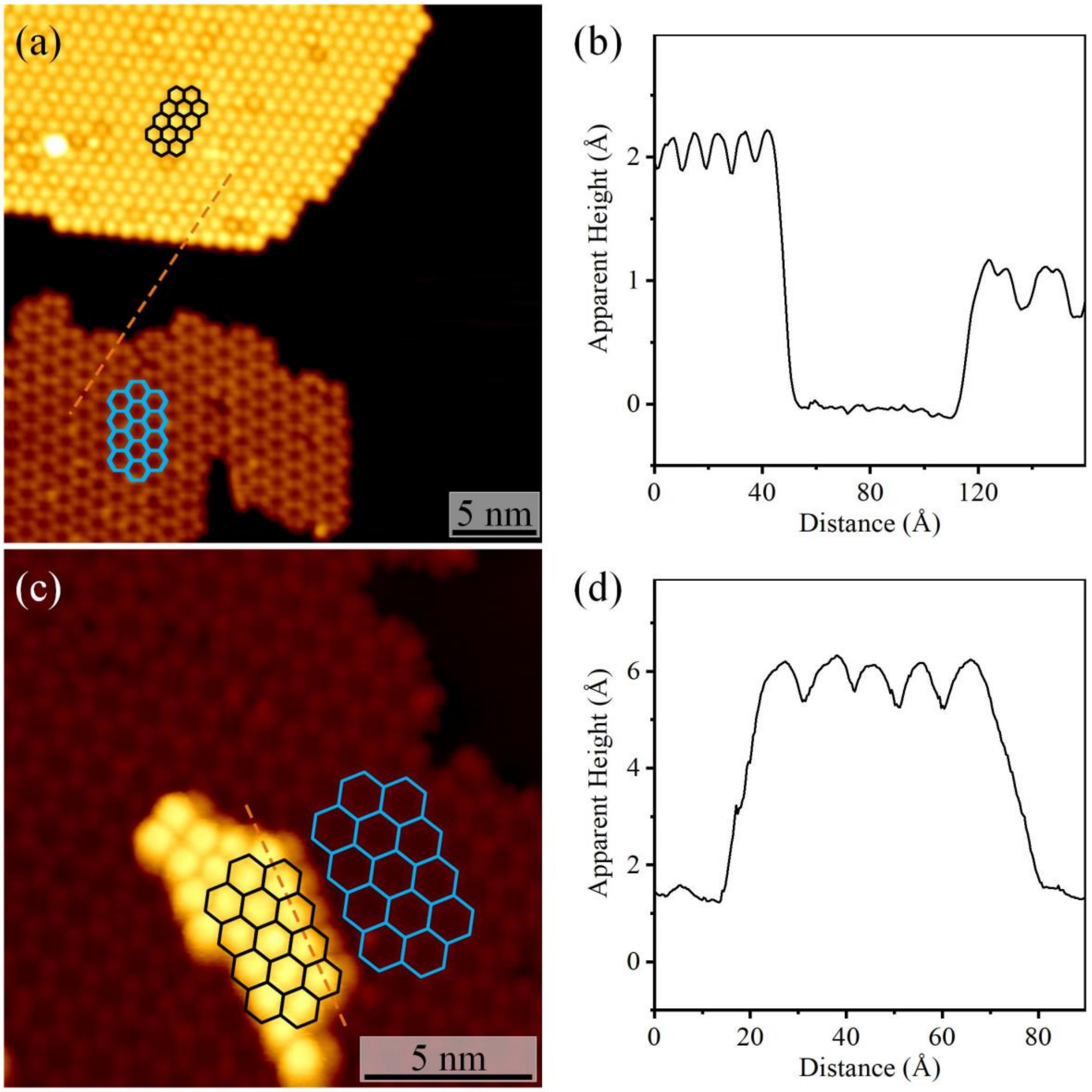

Fig. S9 (a) STM image of a lot of C_{60} molecules adsorbed above Ag(111) surfaces (U = −2.1 V, I = 100 pA), with (b) the height profile along the orange line in Fig. S9a. (c) STM image of C_{60} molecules intensively adsorbed on the H_3btim networks (U = −1.9 V, I = 300 pA), with (d) the height profile along the orange line in Fig. S9c. Black and blue honeycomb-like networks are corresponding to C_{60} accumulation and H_3btim networks.

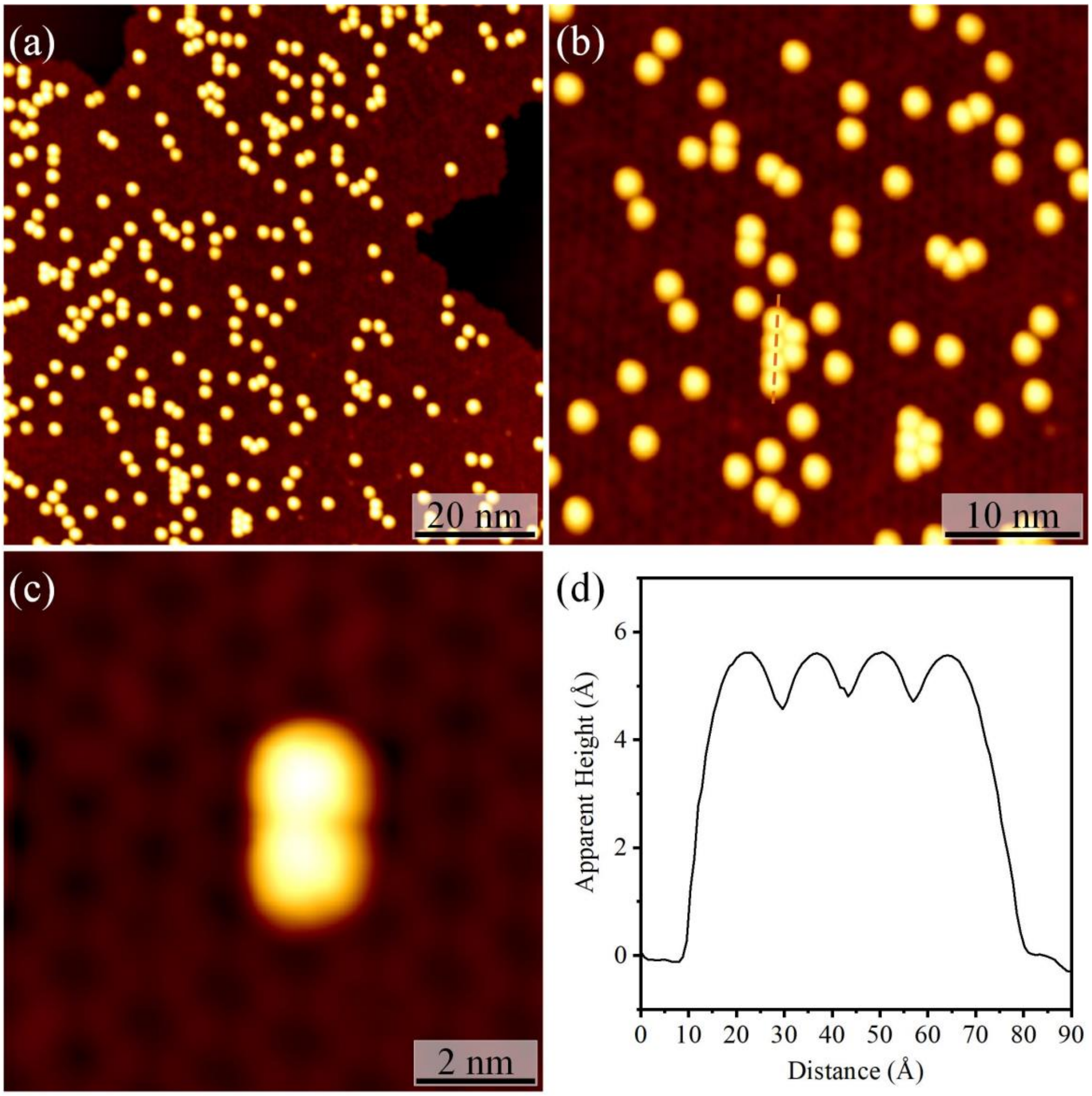

Fig. S10 STM image of C_{60} molecules on the [Ag_3(btim)] in (a) large area (U = 1.8 V, I = 50 pA), and (b) part area (U = 1.5 V, I = 140 pA). (c) High resolution STM image of two C_{60} molecules above the holes of [Ag_3(btim)] (U = 1.7 V, I = 100 pA), and (d) the height profile along the orange line in Fig. S10b.

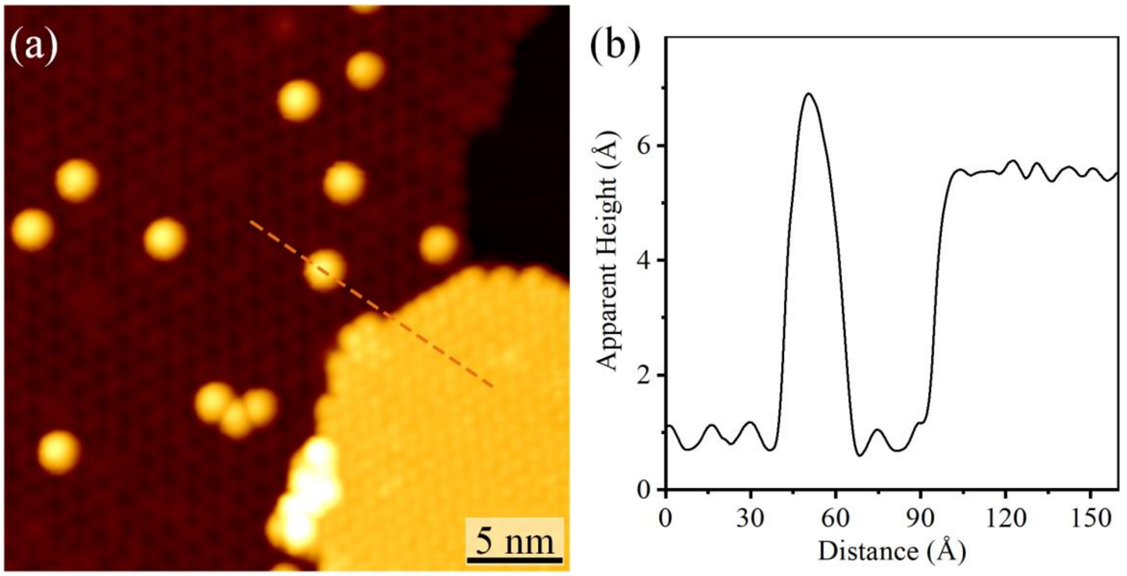

Fig. S11 (a) STM image of C_{60} molecules disperse on the [Ag_3(btim)] network and aggregated on the Ag(111) surfaces simultaneously (U = 1.9 V, I = 50 pA) with (b) the height profile along the orange line in Fig. S11a. The lattice period of [Ag_3(btim)] network and C_{60} aggregation are 14.0 Å and 9.5 Å, respectively.

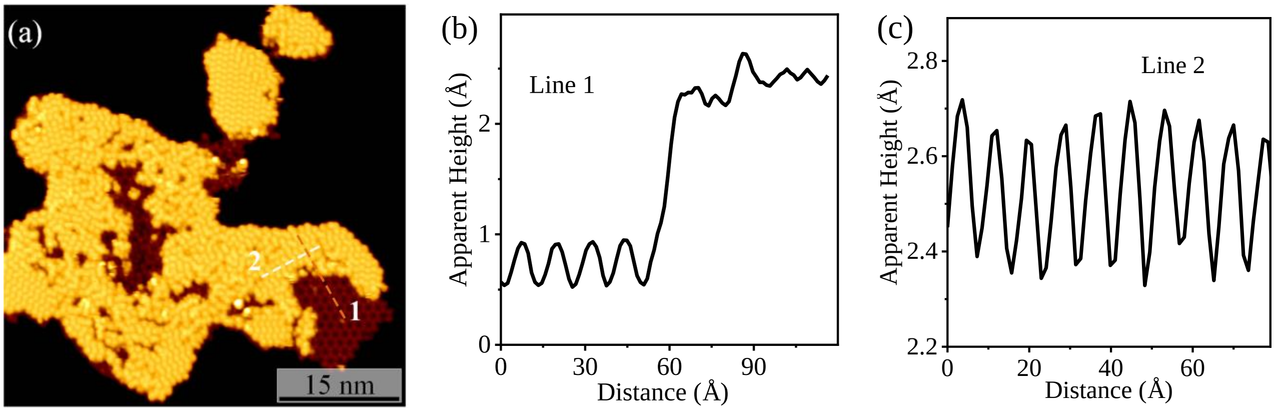

Fig. S12 (a) STM image of $Fe(Cp)_2$ molecules on the H_3btim (U = −3.0 V, I = 10 pA). The height profile along (b) line 1 (orange) and (c) line 2 (white) in Fig. S12a. The lattice period of H_3btim network is 12.1 Å, and $Fe(Cp)_2$ molecules distributed randomly with adjacent distances of 6.3–10.5 Å.

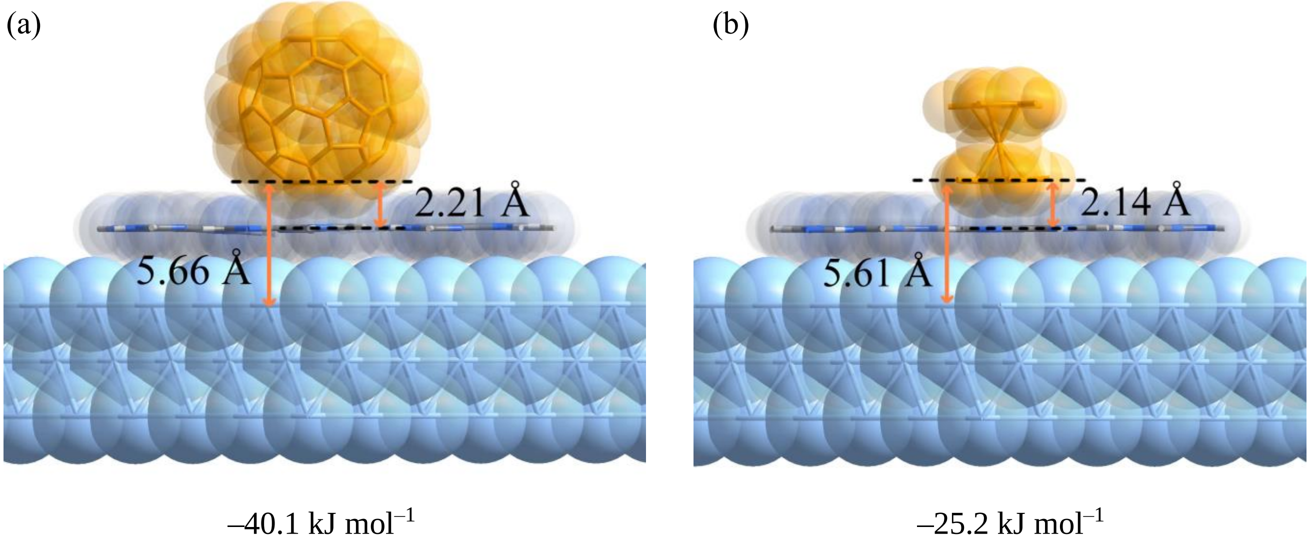

Fig. S13 Side views of (a) C_{60} and (b) $Fe(Cp)_2$ on the hole of H_3btim, obtained by DFT optimizations. The corresponding adsorption enthalpies are marked below.

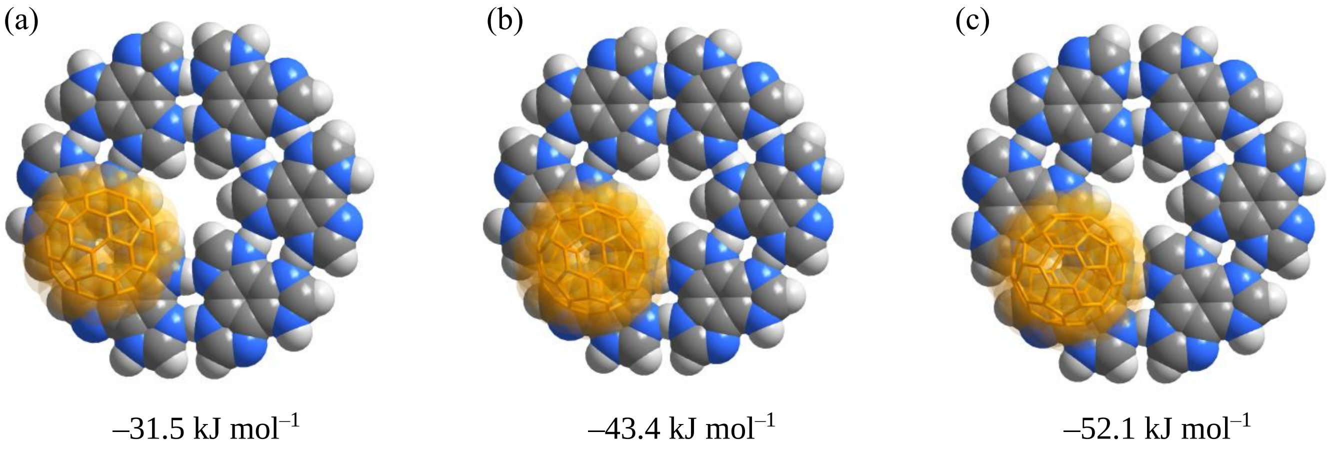

Fig. S14 Top views of C_{60} on (a) hydrogen bonds, (b) phenyl group, and (c) imidazole group of H_3btim, obtained by DFT optimizations. The corresponding adsorption enthalpies are marked below.

(a) (b) (c)

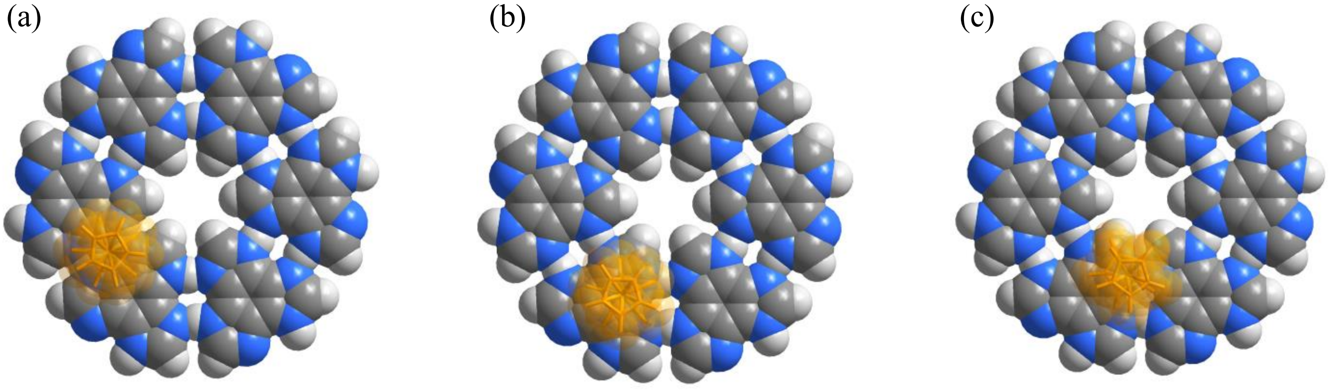

–27.7 kJ mol^{-1} –11.9 kJ mol^{-1} –7.9 kJ mol^{-1}

Fig. S15 Top views of $Fe(Cp)_2$ on (a) hydrogen bonds, (b) phenyl group, and (c) imidazole group of H_3btim, obtained by DFT optimizations. The corresponding adsorption enthalpies are marked below.

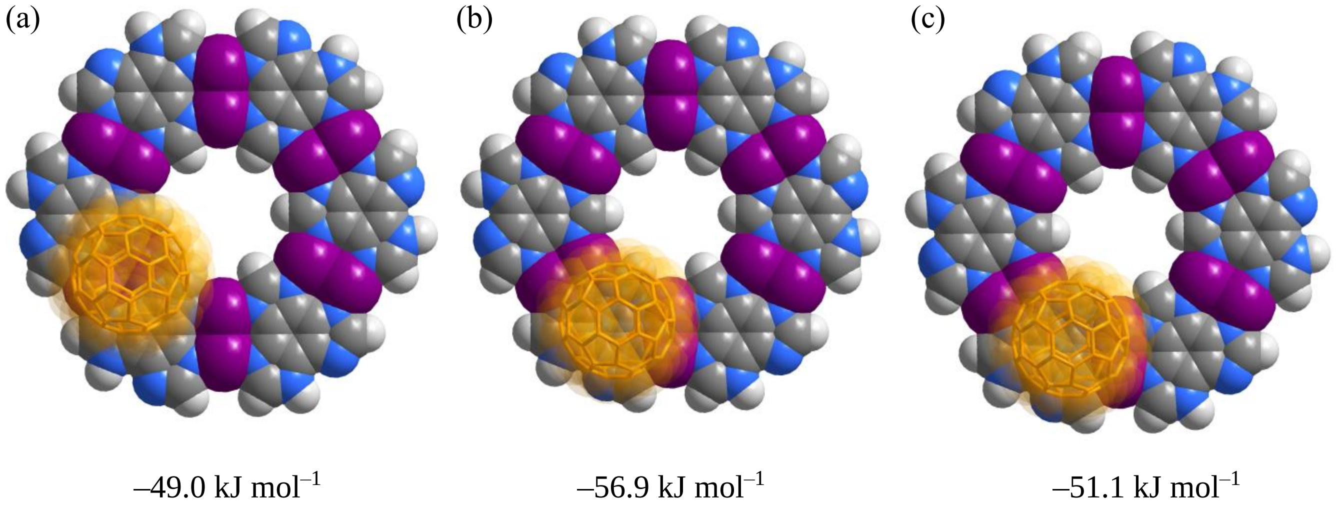

Fig. S16 Top views of C_{60} on (a) double N–Ag–N bonds, (b) phenyl group, and (c) imidazolate group of [Ag_3(btim)], obtained by DFT optimizations. The corresponding adsorption enthalpies are marked below.

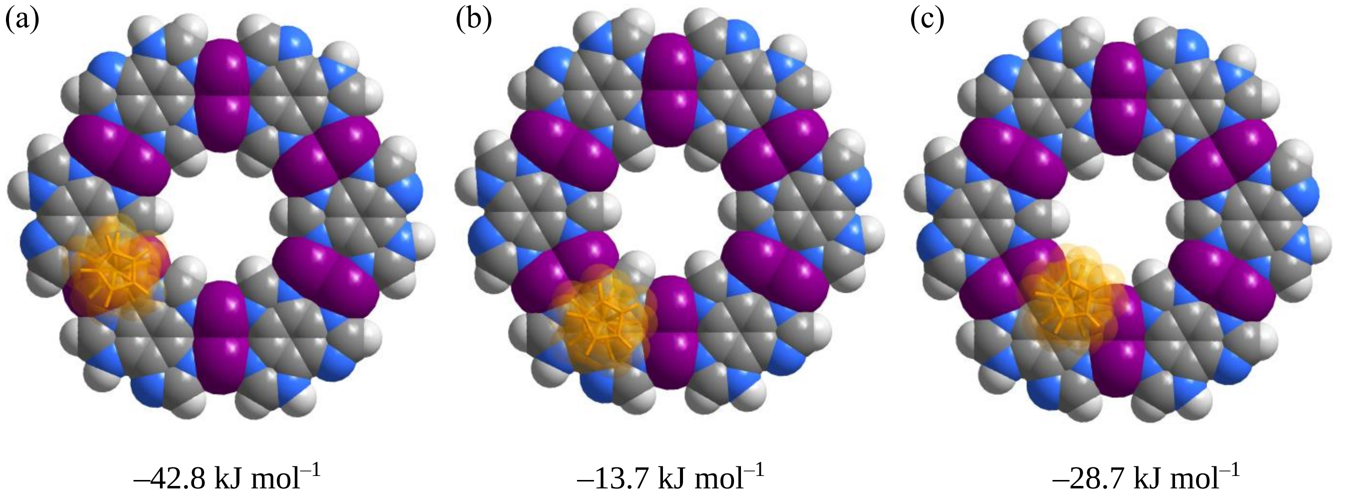

Fig. S17 Top views of $Fe(Cp)_2$ on (a) double N–Ag–N bonds, (b) phenyl group, and (c) imidazolate group of $[Ag_3(btim)]$, obtained by DFT optimizations. The corresponding adsorption enthalpies are marked below.

(a) (b)

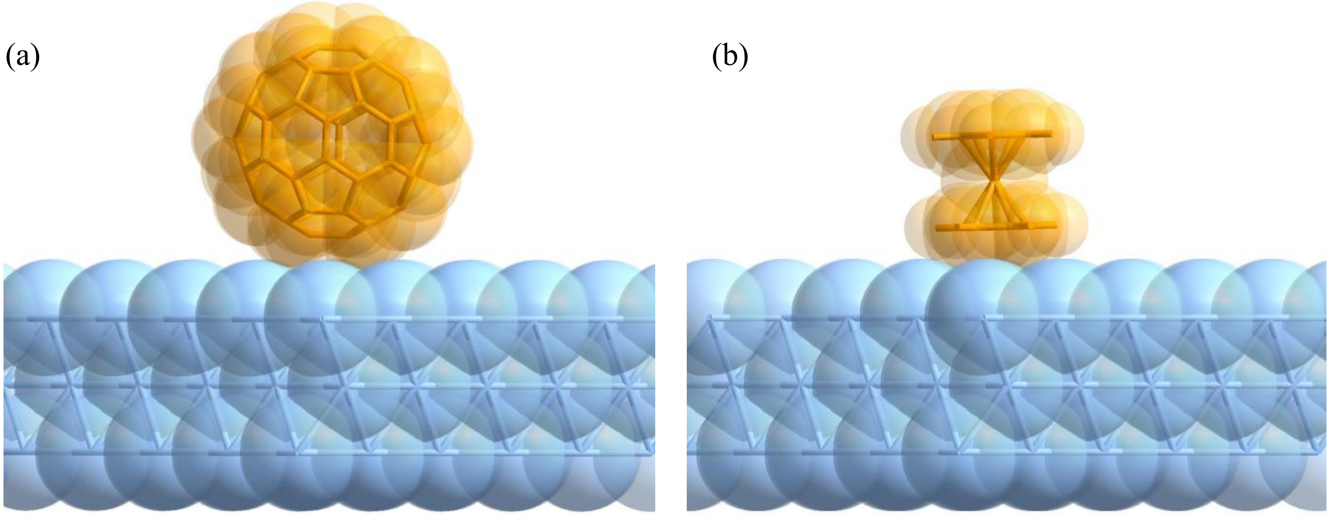

–75.2 kJ mol^{-1} –21.5 kJ mol^{-1}

Fig. S18 Side views of (a) C_{60} and (b) $Fe(Cp)_2$ on Ag(111) surface, obtained by DFT optimizations. The corresponding adsorption enthalpies are marked below.

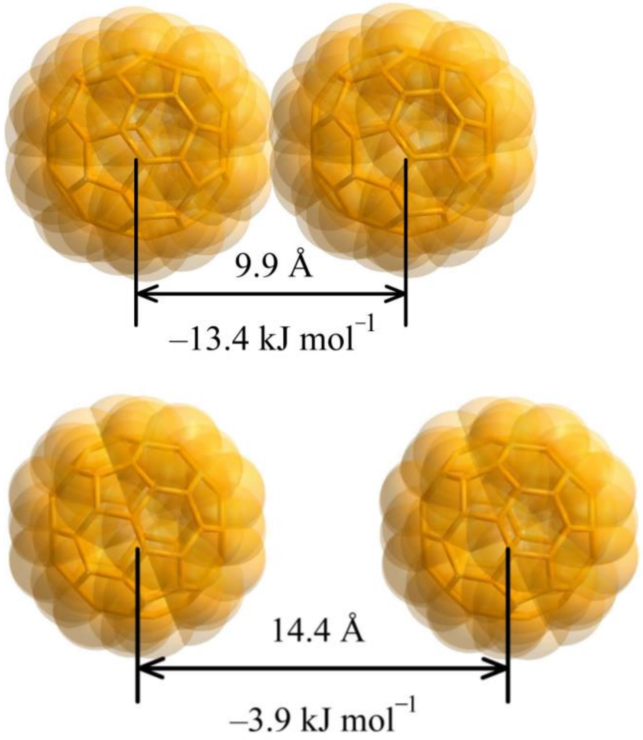

Fig. S19 Guest-guest interactions between two C_{60} molecules with different distances, obtained by DFT optimizations. The corresponding adsorption enthalpies are marked below.
